# Analysis of the Spatiotemporal Heterogeneity and Influencing Factors of Regional Economic Resilience in China

**DOI:** 10.3390/e27010023

**Published:** 2024-12-31

**Authors:** Qiuyue Zhang, Yili Lin, Yu Cao, Long Luo

**Affiliations:** 1 School of Economics and Management, Beijing University of Technology, Beijing 100124, China; zqy@bjut.edu.cn; 2 School of Political Science and Law, University of Jinan, Jinan 250022, China; linyili42@gmail.com; 3 School of Social Research, Renmin University of China, Beijing 100872, China; 4 Department of Physical Education and Research, Central South University, Changsha 410083, China; 8104220108@csu.edu.cn

**Keywords:** economic resilience, entropy method, spatial Durbin model

## Abstract

This study estimates regional economic resilience in China from 2000 to 2022, focusing on economic resistance resilience, recovery resilience, and reorientation resilience. The entropy method, kernel density estimation, and spatial Durbin model are applied to examine the spatiotemporal evolution and influencing factors. The results show significant spatial clustering, with stronger resilience in the east and weaker resilience in the west. While economic resilience has generally improved, regional disparities persist. Key factors such as human capital, urban hospitals, financial development, market consumption, and environmental quality have a positive effect on resilience, with spatial spillover effects. However, human capital and urban hospitals also show a negative indirect impact on surrounding regions. The influence of these factors varies across regions and periods, indicating strong spatiotemporal heterogeneity.

## 1. Introduction

Since China acceded to the World Trade Organization (WTO) in 2001, the country has experienced rapid and sustained economic growth, becoming a key driver of global economic expansion. However, the Chinese economy faces significant challenges both domestically and internationally. Domestically, factors such as a slowdown in labor force growth, shifts in social production environments and consumer demand, and industrial transformation impose new demands on economic development, signaling profound structural changes. Internationally, challenges include the global impact of the COVID-19 pandemic and intensifying geopolitical risks, which increase uncertainties in the global economy and pose unprecedented challenges to China’s economic development.

In this context, regional economic resilience has gained prominence. Initially derived from the concept of “elasticity” in physics, resilience describes a material’s ability to return to its original state after external forces. This concept later expanded to ecology and economics to describe a system’s capacity to recover and adapt following disturbances. Reggiani was among the first to introduce resilience into economics, emphasizing the recovery and adaptability of economic systems after external shocks [1]. Facing challenges from various aspects, enhancing regional economic resilience not only helps ensure economic security but also promotes local industrial optimization and sustained stable economic growth [2]. The Chinese government has emphasized the importance of resilience in key policy documents, such as the 20th National Congress report, the “14th Five-Year Plan”.

Regional economic resilience has become a key topic in regional economics and economic geography. Martin conceptualized regional economic resilience through dimensions such as vulnerability, resistance, stability, and recovery, defining it as an economy’s capacity to resist, recover, and reorganize after shocks [3]. Doran and Fingleton emphasized adaptive resilience, highlighting the ability to adjust during resistance, recovery, and development stages [4]. Maguire and Hagan identified three essential capabilities of resilient systems: resistance, recovery, and creativity [5]. These dimensions allow regions to respond to significant shocks, recover to pre-shock levels, and achieve enhanced functionality.

Building on this literature, this study defines regional economic resilience through three perspectives: economic resistance, recovery, and reorientation resilience. This framework captures an economy’s ability to maintain functionality, recover quickly, and optimize its structure to adapt to new conditions in the face of economic shocks. Previous studies have shown that entropy is an effective measure for capturing economic shocks [6]. Building on this, the present study further applies entropy to comprehensively assess regional economic resilience from the perspectives of resistance, recovery, and reorganization. Using panel data from 31 provinces in China from 2000 to 2022, this study investigates the spatiotemporal evolution of regional economic resilience and the factors influencing its development. Specifically, it seeks to answer the following research questions: (1) How does regional economic resilience evolve over time and space in China? (2) What are the key factors influencing the development of economic resilience in different regions? (3) How do economic, social, and environmental factors interact to shape regional resilience? This study employs the entropy method, kernel density estimation, and map visualization to analyze regional differences and trends in resilience. The spatial Durbin model (SDM) is used to explore the effects of economic, social, and environmental factors on resilience development. The findings aim to provide strategies and policy recommendations for enhancing regional economic resilience in response to the dynamic global economic environment.

The contributions of this study are threefold. First, it provides an innovative multidimensional assessment of how economic, social, and environmental factors comprehensively influence regional economic resilience, filling gaps in the existing literature and offering decision support for policy formulation. Second, by integrating temporal and spatial dimensions into the analytical framework, it employs methods such as kernel density estimation and SDM to reveal the geographic distribution and evolution of resilience, offering a precise scientific basis for regional development strategies. Finally, the study examines the spatiotemporal heterogeneity of influencing factors across provinces and major regions, identifying development potentials and providing insights for building resilient cities in China.

## 2. Literature Review

A review of the existing literature on economic resilience reveals that studies primarily explore three aspects: the measurement methods of economic resilience, the spatiotemporal heterogeneity characteristics of economic resilience, and the analysis of factors influencing economic resilience.

Currently, there is no unified standard for measuring economic resilience within the academic community [7]. Existing measurement methods mainly include the multidimensional index method, core indicator method, counterfactual simulation method, and systemic risk method [8]. The multidimensional index method, first proposed by Briguglio, constructs an index system of economic resilience by combining a series of economic variables that significantly impact economic resilience [9]. This method has been widely adopted by many researchers to assess regional economic resilience [10,11]. The core indicator method selects one or a few variables sensitive to economic shocks to measure economic resilience by calculating the elasticity of these variables before and after a shock, often using employment rates, GDP, or a combination of both for a comprehensive measurement [12,13]. While this method is simple and easy to understand, its drawback lies in its reliance on a single or a few indicators, which may not fully capture the complexity and multidimensional characteristics of economic resilience. Additionally, if the chosen core indicators are not representative enough, it may lead to biased measurement results. The counterfactual simulation method constructs economic models under counterfactual conditions to simulate economic performance without certain shocks, thereby estimating the difference between the fitted values and actual values to assess the real economy’s response and adaptability to shocks [14,15]. However, this method depends on the accuracy of the models and the reasonableness of the assumptions, and any bias in model construction or parameter setting may lead to inaccurate assessment results. Given the increasing global economic risks, the method focusing on systemic risk shocks has also gained attention in academia. This approach, particularly applicable to the financial sector and large-scale economic systems, emphasizes assessing systemic risks within economic systems [16]. However, this method may overlook other types of resilience performance due to its focus on systemic risks. Additionally, it requires a large amount of data and depends heavily on data processing and risk assessment models.

A significant characteristic of economic resilience is its marked spatiotemporal heterogeneity [17]. From the perspective of risk shocks, studies by Capello and Grabner noted that following the 2008 global financial crisis, regional economic resilience exhibited clear spatial differentiation, with large metropolitan areas displaying stronger economic resilience due to their high functional diversity and integration compared to smaller cities [18,19]. However, during the COVID-19 pandemic, studies have shown that economically dense large cities appeared more vulnerable due to the rapid spread and severe impact of the virus, with a slower recovery pace [20,21]. This indicates that economic resilience is not static; different regions often exhibit varying levels of resilience when faced with shocks at different times. Regionally, taking China as an example, the overall regional economic resilience has shown a continuous growth trend, but the gap in economic resilience between regions has persisted over the long term, specifically manifesting as a decrease in regional economic resilience from the eastern coastal areas to the central and western regions, with the economic resilience in southern regions significantly higher than in northern regions [22]. The “clustering” spatial differentiation characteristic of urban resilience is evident [23], with the economic resilience levels of large urban agglomerations significantly higher than other areas, and the Yangtze River Delta urban agglomeration, as a major representative of urban clusters, has consistently exhibited higher economic resilience than other areas, showing a significant positive spatial correlation with a relatively stable spatial agglomeration pattern, and a regional internal distribution pattern of “high in the east, low on the northern and southern edges” [24].

Regarding the factors influencing economic resilience, existing studies have mainly elucidated aspects such as industrial structure, human capital, financial conditions, openness, and infrastructure construction. Specifically, industrial diversification is one of the key factors enhancing economic resilience, as noted by Brown and Greenbaum, who pointed out that industrial diversification helps regional economies withstand external shocks by dispersing risks within economic activities [25]. Human capital is also an important factor affecting urban economic resilience; increasing urban population density and enhancing urban human capital are effective measures to strengthen urban resilience [26]. The financial industry significantly enhances economic resilience by injecting vitality into innovation and entrepreneurship, accelerating industrial structure upgrading, and thereby improving economic resilience [27]. In the context of economic globalization, the level of market openness has a significant impact on regional economic resilience, and promoting market openness during economic downturns has a more pronounced effect on enhancing urban economic resilience [28]. Infrastructure construction is a major pillar of economic resilience; improved infrastructure not only enhances regional connectivity but also increases the efficiency of economic activities. Research by Zhang and Hui, based on China’s agricultural data, indicated that infrastructure construction is a key factor in enhancing economic resilience [29]. Other studies have explored economic resilience from perspectives such as innovation capacity [30], urban environmental construction [31], fixed asset investment [32], and government intervention [33], but most research tends to analyze the impact of specific factors from a single perspective, overlooking the potential interactions and synergistic effects among different factors.

In the process of measuring economic resilience and analyzing its influencing factors, although traditional methods have provided a multi-dimensional framework for understanding, they may have limitations when faced with the complex nonlinear relationships and dynamic changes within economic systems. This is particularly true during economic crises or extreme events, when the behavior of the economic system is often characterized by uncertainty. In such cases, more precise and flexible analytical tools become especially important. Entropy methods, as tools for measuring system complexity and information flow, can effectively complement traditional measurement approaches, offering unique advantages in revealing nonlinear relationships in areas such as economic resilience and market efficiency. For instance, van Kralingen et al. explored the impact of market clustering on stock price stability and found that crowded trading, via the maximum entropy concept, significantly affects the tail distribution of stock returns [34]. Olbryś and Ostrowski proposed an entropy-based approach for measuring stock market depth, providing a new framework for analyzing market liquidity [35]. Liao et al. applied entropy and network methods to examine risk spillover effects in China’s bond market, revealing the transmission pathways of risk between different bond types [36]. Additionally, Będowska-Sójka et al. studied the relationships between prices, volatility, and liquidity in Bitcoin and other cryptocurrencies, using transfer entropy to analyze the directional flow of information in cryptocurrency markets [37]. In the study of market efficiency, entropy methods also play a crucial role. For example, Olbryś and Komar employed symbolic encoding and modified Shannon entropy to assess market efficiency, revealing a significant decline in entropy during extreme events such as the COVID-19 pandemic and the Ukraine war, indicating a decrease in market informational efficiency during crises [38]. Similarly, Papla and Siedlecki used Shannon entropy to analyze stock market efficiency during financial crises, pandemics, and wars, confirming a significant reduction in market entropy during periods of economic downturn and crisis [39]. These studies demonstrate that entropy methods not only uncover the complexity of financial markets but also provide fresh perspectives and theoretical support for understanding regional economic resilience and market efficiency, thus contributing to innovations in economics, finance, and management.

In summary, this paper, based on existing research on economic resilience, proposes a multidimensional assessment model that comprehensively considers economic, social, and environmental factors. This model’s innovative aspect lies in enhancing the comprehensiveness and depth of analysis of factors affecting economic resilience and revealing the dynamic changes and regional differences in economic resilience through the comprehensive application of spatiotemporal data.

## 3. Research Design

### 3.1. Data Sources

The data for calculating regional economic resilience and its influencing factors are sourced from the “China Statistical Yearbook”. To eliminate the impact of exchange rate fluctuations, the portion involving foreign capital has been converted into RMB using the average exchange rate for the respective years. Missing data were filled using interpolation methods.

### 3.2. Construction of the Economic Resilience Index System

Existing studies have not yet reached a consensus on the measurement of economic resilience. As previously mentioned, depending on the focus of the research, current assessment methods primarily include the multidimensional index method, core indicator method, counterfactual simulation method, and systemic risk method. After a comprehensive comparison of the advantages and disadvantages of these methods and the availability of data, this study ultimately chose to use the multidimensional index method to measure regional economic resilience. This method can comprehensively reflect the multidimensional characteristics of economic resilience, ensuring the scientific accuracy of the research results to the greatest extent. Compared to the single indicator method and counterfactual estimation, the multidimensional index method integrates multiple indicators, such as the level of economic development, industrial structure, and local development capabilities, to fully assess the economy’s performance in three key dimensions: resistance, recovery, and reorganization [40]. This method not only captures the complexity and multidimensional characteristics of economic resilience but also reduces biases in the assessment results caused by the selection of a single indicator.

In terms of specific indicator selection, based on the definition of economic resilience, economic resistance resilience emphasizes an economy’s ability to withstand external risk shocks, which is closely related to its existing economic foundation [41]; economic recovery resilience emphasizes an economy’s ability to recover after experiencing risk shocks, mainly including the optimization of economic structure and allocation of resources [42,43]; economic reorganization resilience emphasizes an economy’s ability to develop after experiencing shocks, covering regional innovation and future development [44,45]. Specific indicators are shown in Table 1.

### 3.3. Research Methods

#### 3.3.1. Entropy Method

In constructing a composite index system to assess regional economic resilience, a key step is determining the weights of the indicators within the system. Generally, methods for determining weights can be divided into subjective weighting and objective weighting methods. Subjective weighting methods, such as the Delphi method and analytic hierarchy process (AHP), rely on the subjective judgments of experts to set weights. While these methods can reflect experts’ experience and insights, they are susceptible to individual subjective biases. In contrast, objective weighting methods, like cluster analysis and the entropy method, determine weights based on the statistical characteristics of the data, effectively reducing the impact of human subjective interference and making the weight setting more objective and scientific.

The entropy method, as an objective weighting approach, is rooted in the concept of information entropy from information theory, which differs from thermodynamic entropy in physics. Information entropy refers to the amount of information required to accurately send and receive a message [46]. While thermodynamic entropy measures the degree of disorder or randomness in a physical system, information entropy quantifies the amount of information or uncertainty needed to describe the state of a system. In economic analysis, information entropy holds particular value, as it provides an efficient way to measure risk and volatility, especially when return data do not follow a Gaussian distribution [47]. For instance, entropy has been shown to replace standard deviation as a measure of volatility [48] and is also highly effective in capturing economic shocks [6] and calculating risk [49]. It has also been applied to describe correlation-based networks in economic systems [50,51], and information entropy is especially significant in financial studies where traditional models may not apply [46].

To ensure the comprehensiveness and practicality of the evaluation, this study uses the entropy method to calculate weights and has normalized indicators of different levels, dimensions, and magnitudes. This step is not only necessary foundational work for constructing a composite index system but also ensures the horizontal comparability among indicators, making the final composite index more scientific and operable.

- Step One: Normalization of Indicators

During the normalization process, it is essential to accurately distinguish the direction of the indicators to ensure that each indicator correctly reflects its impact on the system in the overall evaluation.

Positive Indicators: For indicators where a higher value is more beneficial for the development of the system, the normalization is processed using the following formula:

(1) $$x_{ij}=\frac{x_{ij}-\min\left(x_{j}\right)}{\max\left(x_{j}\right)-\min\left(x_{j}\right)}$$

Negative Indicators: For indicators where a lower value is more beneficial for the development of the system, the normalization is processed using the following formula:

(2) $$x_{ij}=\frac{\max\left(x_{j}\right)-x_{ij}}{\max\left(x_{j}\right)-\min\left(x_{j}\right)}$$

Here, $x_{ij}$ is the original value of the i observation for the j indicator, $\max\left(x_{j}\right)$ and $\min\left(x_{j}\right)$ are the maximum and minimum values of the indicator across all observations, respectively.

- Step Two: Calculation of Information Entropy

The entropy value $e_{j}$ for the j indicator is calculated using the following formula:

(3) $$e_{j}=-\frac{1}{\ln\left(n\right)}\overset{n}{\underset{i=1}{\sum }}p_{ij}\ln\left(p_{ij}\right)$$

where $p_{ij}=\frac{x_{ij}}{\sum_{i-1}^{n}x_{ij}}$, and n is the sample size. If $p_{ij}=0$, then $p_{ij}\ln\left(p_{ij}\right)=0$. The entropy value $e_{j}$ ranges from 0 to 1.

- Step Three: Calculation of Entropy Weights

The entropy weight of an indicator reflects the degree of variation in the information entropy and is used to determine the weight of each indicator. The formula for calculating the entropy weight $w_{j}$ is as follows:

(4) $$w_{j}=\frac{1-e_{j}}{m-\sum_{j=1}^{m}e_{j}}$$

where m is the total number of indicators, and $1-e_{j}$ represents the redundancy of information entropy for the j indicator.

- Final Step: Calculation of the Composite Score

Using the entropy weights and the standardized data, the composite score for each evaluation object is calculated as follows:

(5) $$S_{i}=\overset{m}{\underset{j=1}{\sum }}w_{j}x_{ij}$$

where $S_{i}$ is the composite score of the i evaluation object, and m is the total number of indicators selected across all levels in the evaluation system.

#### 3.3.2. Moran’s Index

In this study, Moran’s index is used to test for spatial correlation in the level of regional economic resilience across the 31 provinces of China, which includes both global and local correlations. The specific formulae are as follows:

Global Moran’s Index:

(6) $$I=\frac{n\sum_{i=1}^{n}\sum_{j=1}^{n}w_{ij}\left(x_{i}-\bar{x}\right)\left(x_{j}-\bar{x}\right)}{\sum_{i=1}^{n}\sum_{j=1}^{n}w_{ij}\sum_{i=1}^{n}{\left(x_{i}-\bar{x}\right)}^{2}}$$

where *n* is the total number of observation regions, $x_{i}$ and $x_{j}$ are the attribute values of observation regions i and j, respectively, $\bar{x}$ is the mean of all observations, and $w_{ij}$ is the spatial weight matrix, representing the spatial proximity between two regions.

Local Moran’s Index:

(7) $$I_{i}=\frac{\left(x_{i}-\bar{x}\right)\sum_{j=1}^{n}w_{ij}\left(x_{j}-\bar{x}\right)}{\sum_{i=1}^{n}{\left(x_{i}-\bar{x}\right)}^{2}}$$

$I_{i}$ represents the local Moran’s index for region i.

#### 3.3.3. Kernel Density Curve

Kernel density estimation is a nonparametric method for estimating probability distributions solely based on the characteristics of the data. It has advantages such as low dependence on the model and strong robustness [52]. In this study, the Gaussian kernel density function is used to dynamically estimate the distribution of regional economic resilience levels in Chinese provinces from 2000 to 2022. The specific formula is as follows:

(8) $$\hat{f}\left(x\right)=\frac{1}{nh}\overset{n}{\underset{i=1}{\sum }}K\left(\frac{x-x_{i}}{h}\right)$$

where $\hat{f}\left(x\right)$ is the estimated density at point x, n is the number of samples, $x_{i}$ is the i data point, h is the bandwidth that controls the smoothness of the estimate, and K() is the kernel function that controls the contribution of each point $x_{i}$ to the estimation results. Considering the simplicity of the Gaussian kernel function, its ease of use, and its good smoothness, this paper opts for the Gaussian kernel function for kernel density estimation. The formula for the Gaussian kernel function is as follows:

(9) $$K\left(u\right)=\frac{1}{\sqrt{2\pi }}e^{-\frac{u^{2}}{2}}$$

Substituting Equation (9) into Equation (8) provides the probability density function:

(10) $$\hat{f}\left(x\right)=\frac{1}{nh}\overset{n}{\underset{i=1}{\sum }}\frac{1}{\sqrt{2\pi }}e^{-\frac{1}{2}{\left(\frac{x-x_{i}}{h}\right)}^{2}}$$

#### 3.3.4. Spatial Durbin Model

In regional economic analysis, considering spatial correlations is crucial because the economic performance of an area can be influenced by its neighboring regions. To accurately capture these spatial interactions, the SDM was developed. It takes into account the spatial lag effects of both dependent and independent variables. By employing this method, the SDM can comprehensively analyze and explain the direct and indirect impacts of explanatory variables and their spatial lags on the dependent variable, providing a comprehensive understanding of the spatial data structure. The specific expressions are as follows:

(11) $$res_{it}=\delta_{1}\overset{n}{\underset{j=1}{\sum }}W_{ij}rer_{jt}+\beta_{1}hcl+\beta_{2}fdl+\beta_{3}mcl+\beta_{4}mol+\beta_{5}uhl+\beta_{6}ueq+\;\;\;\;\;\;\;\theta_{1}\overset{n}{\underset{j=1}{\sum }}W_{ij}hcl_{jt}+\theta_{1}\overset{n}{\underset{j=1}{\sum }}W_{ij}fdl_{jt}+\theta_{1}\overset{n}{\underset{j=1}{\sum }}W_{ij}mcl_{jt}+\theta_{1}\overset{n}{\underset{j=1}{\sum }}W_{ij}mol_{jt}+\;\theta_{1}\overset{n}{\underset{j=1}{\sum }}W_{ij}uhl_{jt}+\theta_{1}\overset{n}{\underset{j=1}{\sum }}W_{ij}ueq_{jt}+\mu_{it}+\varepsilon_{it}$$

where i represents the province, t represents the year, j represents neighboring provinces (where j≠i), res represents regional economic resilience, hcl represents human capital level, fdl represents financial development level, mcl represents market consumption level, mol represents investment openness level, uhl represents urban hospital level, ueq represents urban environmental quality, W is the spatial weight matrix, δ is the spatial autoregressive coefficient of the independent variables, β are the regression coefficients for the dependent variable, θ are coefficients for spatial interaction terms of independent variables, μ represents fixed effects, and ε represents random disturbance terms. This paper constructs a geographic adjacency matrix and a geographic distance matrix to reflect spatial correlations.

### 3.4. Selection and Explanation of Influencing Factors

In selecting the influencing factors for economic resilience, it is crucial to adopt a unified and objective set of indicators, given the significant economic and cultural differences across regions in China. The advantage of this approach lies in its ability to avoid indicator bias caused by regional characteristics, thus providing a more accurate reflection of overall economic resilience. First, human capital is often seen as a key driver of economic development and resilience. High levels of human capital not only promote technological advancement but also enhance the ability to cope with economic crises, thereby strengthening economic resilience [53,54]. Second, financial development and market consumption play a crucial role in assessing regional economic stability and adaptability, particularly in the context of rapid urbanization and industrialization. A sound financial system and active market consumption not only enhance economic resilience but also provide essential support for regional development [55,56]. Investment openness reflects the degree to which regions are integrated into the global economy and its impact on economic flexibility [21]. Healthcare levels and environmental quality reflect the growing recognition of the role of public health and environmental factors in economic resilience, particularly after the outbreak of the COVID-19 pandemic [57,58,59]. The inclusion of these variables illustrates the interplay between economic, social, and environmental factors (See Table 2 for details).

Descriptive statistics for each variable are presented in Table 3.

## 4. Empirical Analysis

### 4.1. Analysis of the Spatiotemporal Evolution Characteristics of Economic Resilience

#### 4.1.1. Global Spatial Autocorrelation

As indicated by Table 4, from 2000 to 2020, the global Moran’s I indices were all greater than zero and significantly rejected the null hypothesis at the 1% level, indicating a positive spatial correlation in China’s economic resilience. This means that the economic resilience levels of provinces are closely related to those of their neighboring areas, with high-resilience regions surrounded by other high-resilience regions (H-H type) and low-resilience regions surrounded by other low-resilience regions (L-L type). This demonstrates that China’s economic resilience exhibits spatial clustering characteristics, aligning with the conclusions of existing research [60].

From a temporal perspective, the global Moran’s I indices generally show an upward trend, increasing from 0.340 in 2000 to 0.392 in 2022, suggesting that the spatial correlation in economic resilience among provinces and cities is gradually strengthening. Specifically, the global Moran’s I indices have displayed a fluctuating upward trend and experienced two significant inflection points in 2008 and 2015. The global financial crisis in 2008 led to a trough in the global Moran’s I index. Subsequently, the Chinese government implemented a series of economic stimulus measures to boost domestic demand and investment. These policies, to some extent, accelerated the synchronization of economic activities between regions and enhanced the spatial correlation of economic resilience. The year 2015 correlates with the summer stock market crisis, the effects of which persisted until 2020. During this period, influenced by industrial transformation and upgrading policies, the global Moran’s I index remained at a low level until it finally reached a historical high in 2021.

#### 4.1.2. Local Spatial Autocorrelation

**Figure 1:**
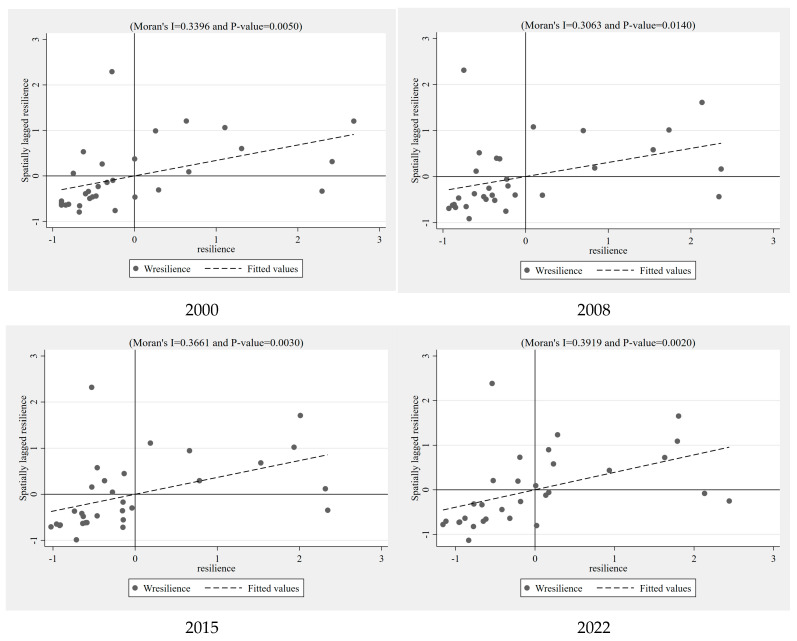
Moran scatter plot (scatter plot of regional economic resilience for selected years in China under adjacency matrix).

Further analysis through scatter plots reveals the spatial association characteristics of economic resilience levels across different provinces. This study selects four-time points—2000, 2008, 2015, and 2022—to conduct an in-depth analysis of the local spatial autocorrelation of regional economic resilience in China. As illustrated in Figure 1, consistent with the above analysis, most Chinese provinces are located in the first and third quadrants, exhibiting high–high (H-H) and low–low (L-L) spatial association patterns, respectively. Specifically, from a spatial distribution perspective, most eastern coastal provinces are located in the first quadrant, indicating that these areas not only possess high economic resilience themselves but also display similar high economic resilience characteristics with neighboring provinces. In contrast, provinces in central and western China are mainly concentrated in the third quadrant, indicating these areas have lower economic resilience, and they also exhibit similar low economic resilience traits with neighboring provinces. Only a few provinces are located in the second and fourth quadrants. This distribution pattern reveals the unevenness of China’s regional economic development, especially the stark contrast between the developed eastern provinces and the less developed central and western provinces. Eastern provinces started economic development earlier, accumulating a wealth of talents, capital, and technology, boasting a more mature industrial base, stronger innovation capabilities, and more developed infrastructure. Meanwhile, provinces in central and western China have weaker economic foundations, started development later, and possess a more homogenous economic structure with less industrial diversity, thus exhibiting significant differences in economic resilience. Additionally, this spatial distribution characteristic also reflects the effects and limitations of regional economic integration policies. Although the government has promoted a series of policies aimed at balanced regional economic development, such as developing the western regions and revitalizing the old industrial bases of the northeast, the actual effects of these policies still show heterogeneity across different regions, such as the entire Yangtze River Delta region being in the first quadrant, while only Guangdong in the Pearl River Delta is in the fourth quadrant, with Guangxi and Hainan in the second quadrant. Therefore, future regional development strategies need to focus more on inter-regional economic coordination and optimal resource allocation to narrow the gap in economic resilience among provinces and promote overall economic resilience and sustainable development.

From a temporal perspective, the development trajectories of different provinces over the years also show significant differences. Taking the Beijing–Tianjin–Hebei urban agglomeration as an example, Beijing’s development trend has gradually shifted from the first quadrant to the fourth quadrant, indicating that although Beijing’s economic resilience remains high, its economic resilience correlation with neighboring areas is weakening. In contrast, Tianjin continues to move leftward within the first quadrant, meaning that while it maintains similar high economic resilience with areas like Beijing, its own economic resilience is showing some weakening. Hebei has fallen from the first to the second quadrant, reflecting a disconnection in economic resilience with neighboring areas and a decline in its own resilience, demonstrating uncoordinated and uneven development within the Beijing–Tianjin–Hebei urban cluster. In the northeast, Liaoning has gradually fallen from the fourth to the third quadrant, indicating that the development of the northeast still requires sustained effort. In the central region, Anhui has made particularly notable progress, jumping from the second to the first quadrant, indicating that the province has not only strengthened its own economic resilience but also formed a closer correlation with economically stronger neighboring provinces. This change could be due to the spillover effects of economic activities from coastal areas and Anhui’s achievements in attracting investment and improving industrial structure.

#### 4.1.3. Spatiotemporal Evolution of Economic Resilience

**Figure 2:**
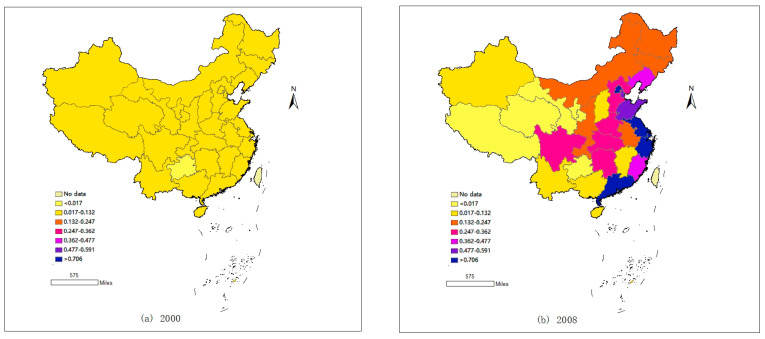

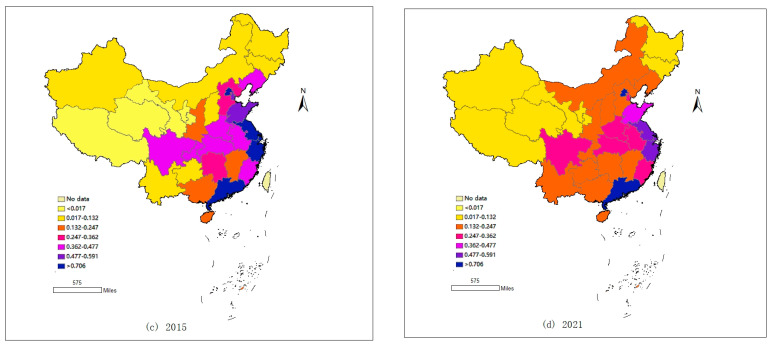
Spatiotemporal evolution of regional economic resilience in China.

To visually represent the spatiotemporal trends in regional economic resilience across Chinese provinces, this study employs the natural breaks (Jenks) classification method to divide the development levels of economic resilience into seven categories from 2000 to 2022. Additionally, using ArcGIS software 10.6, spatial distribution maps of the economic resilience levels of China’s 31 provinces for the years 2000, 2008, 2015, and 2021 were created (see Figure 2).

Figure 2 shows that the overall regional economic resilience levels of Chinese provinces exhibit a gradient decline from east to middle to west, with the eastern coastal regions exhibiting the highest levels of economic resilience, the central regions being at a moderate level, and the western regions being the weakest, showing clear spatial differentiation. In 2000, the level of economic resilience across Chinese provinces was relatively low, with Guizhou being a particularly weak area, indicating that at that time, China as a whole was less resistant to the global financial crisis and highly susceptible to external environmental impacts. By 2008, China’s overall economic resilience showed an upward trend, with the fastest rise in the eastern regions, particularly noticeable in the Yangtze River Delta and Beijing and Guangdong. The central and northeastern regions also experienced some development, although some western provinces saw a decline, highlighting that since joining the WTO, not only has China’s economy grown rapidly but its economic resilience has also strengthened, especially in the foreign trade-driven eastern coastal regions. By 2015, regional economic resilience in China had further developed. While maintaining high levels in the eastern coastal regions, the resilience levels in the central regions also significantly improved, especially noticeable along the Yangtze River economic belt. However, economic resilience in the northeastern regions showed a decline, closely related to national policies implemented since the 18th National Congress aimed at eliminating outdated production capacities, optimizing industrial structures, and advancing the digital transformation of industries. By 2021, the overall structure of regional economic resilience in China had undergone significant changes. Affected by factors such as the COVID-19 pandemic, the overall level of economic resilience in the eastern coastal regions showed a noticeable decline, with only Guangzhou maintaining a high level. The overall economic resilience along the Yangtze River economic belt also showed a downward trend, but economic resilience in other central regions and some western areas exhibited an upward trend, closely related to the implementation of China’s strategy of fostering a strong domestic circulation.

#### 4.1.4. Dynamic Evolution Analysis of Economic Resilience

**Figure 3:**
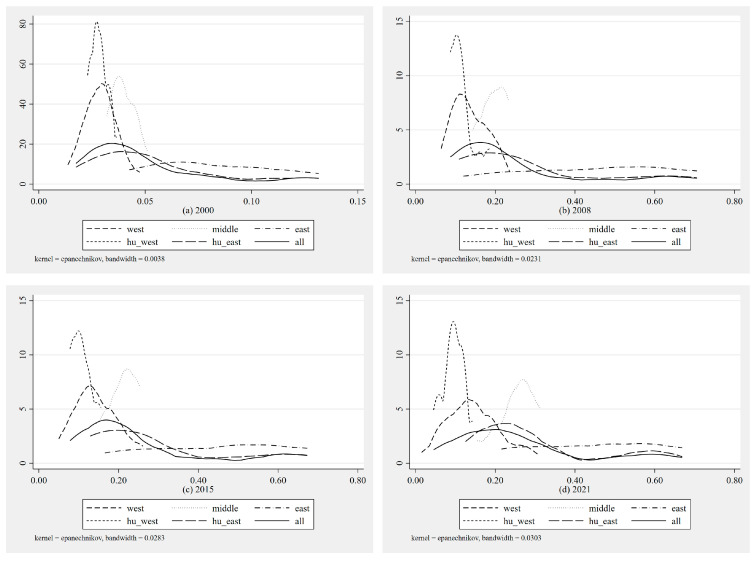
Dynamic distribution of national and major regional economic resilience (kernel density plot).

This paper employs the kernel density estimation method to analyze the dynamic distribution of regional economic resilience development in China. Drawing on the research by Yang et al. [61], this study categorizes the regions of China according to the east, central, and west divisions, as well as by the Hu Huanyong Line. Figure 3 presents the kernel density distribution dynamics of economic resilience across these regions. From Figure 3, it is evident that from 2000 to 2008, the overall national and regional economic resilience distribution curves show a clear rightward shift over time, reflecting the development process from a lower to a higher level of economic resilience. After 2008, the development pace slowed, and most areas exhibited a “single peak” characteristic, which needs to be discussed by region. Starting with the western regions, the peak value of economic resilience consistently remained around 0.15, indicating little overall change in economic resilience, but an increasingly apparent right tail suggests that the maximum economic resilience in the western regions continues to rise. In the central regions, the economic resilience curve noticeably shifted to the right over time, indicating a continuous improvement in the overall economic resilience. For the eastern regions, the overall economic resilience curve remained almost parallel to the *X*-axis, with the left end continuously moving rightward, showing that the minimum economic resilience in the eastern regions is continually increasing. In the areas west of the Hu Huanyong Line, overall economic resilience remained weak and did not show significant growth, with a noticeable rightward shift of the left end following the impact of the COVID-19 pandemic in 2021; in contrast, areas east of the Hu Huanyong Line largely mirrored the national trend, with a more apparent right tail. In summary, there are certain differences in the level of regional economic resilience development among provinces in China, with the eastern regions noticeably outperforming other areas.

### 4.2. Analysis of Factors Affecting Economic Resilience

#### 4.2.1. Suitability Test for Spatial Panel Models

Previous results from Moran’s I test confirmed the presence of spatial autocorrelation, suggesting the necessity of constructing a spatial econometric model. However, the current data do not directly determine the specific form of the model; thus, further model diagnostic tests are needed to select an appropriate model structure. The specific diagnostic results are shown in Table 5.

From Table 5, the LM tests (LM-test-lag, LM-test-error, Robust LM-test-lag, and Robust LM-test-error) are all significant at the 1% level, indicating the simultaneous existence of spatial lag effects and spatial error effects. Therefore, the SDM is considered. The LR and Wald tests, used to assess whether the SDM could degenerate into a spatial lag model (SAR) or spatial error model (SEM), are also significant at the 1% level, rejecting the null hypothesis that SDM degenerates into SAR or SEM, indicating that SDM fits the data better. In terms of fixed effects tests, SDM with individual fixed effects, time fixed effects, and both fixed effects are established and tested through likelihood ratio tests. The results, significant at the 1% level for both LR-both/ind and LR-both/time, indicate that employing both fixed effects is more effective when choosing SDM. Finally, the Hausman test results recommend using a fixed effect model, concluding that a fixed effect SDM should be chosen for the analysis.

#### 4.2.2. Regression Results and Analysis

Table 6 displays the regression results for the OLS model, the adjacency matrix-based SDM, and the geographic distance matrix-based SDM. The spatial regression coefficient rho in the SDM is significantly positive, indicating a spatial spillover effect among the provinces of China, i.e., an increase in economic resilience in neighboring provinces significantly enhances the economic resilience level of the province itself.

SDM regression results are not directly used to discuss the marginal effects of various factors on economic resilience. Following LeSage and Pace’s recommendation [62], a partial differentiation approach is employed to decompose the SDM regression coefficients into total effects, direct effects, and indirect effects. The specific decomposition results are presented in Table 7.

In the decomposition of spatial effects, direct effects represent the impact of the independent variables on the economic resilience level within the region itself, while indirect effects represent the impact on neighboring regions’ economic resilience. From Table 7, using the adjacency matrix as an example:

(1) Human Capital: An increase significantly enhances local economic resilience, reflecting the direct positive impact of human capital on improving productivity and innovation capabilities. However, its indirect effect is significantly negative, suggesting that high human capital regions may act as a resource attraction center for surrounding areas, thereby “siphoning off” economic resilience from neighboring areas.
(2) Financial Development: Both direct and indirect effects are significantly positive, indicating that a mature financial system not only directly enhances the economic stability and growth potential of the local area but also strengthens the economic resilience of surrounding areas through capital flows and regional spread of financial services. This positive spillover effect highlights the role of financial interconnectedness in promoting regional economic integration.
(3) Market Consumption: Growth directly enhances the economic resilience of the area, reflecting the importance of market consumption in strengthening supply-side structures and stimulating consumer demand. Market consumption growth also has a significant positive indirect effect on neighboring areas, suggesting that a developed market can promote local economic growth and drive economic vitality in surrounding areas through trade in goods and services and diffusion of knowledge and technology.
(4) Investment Openness: Neither direct nor indirect effects are significant, indicating that although regional open policies aim to attract foreign investment to boost economic development, due to the balanced implementation of policies and insufficient utilization of foreign capital in key areas, the expected positive impact on economic resilience has not materialized.
(5) Urban Hospital: Improvement significantly enhances local economic resilience, mainly reflected in the optimization of medical resources and an increase in health capital. However, the indirect effect is significantly negative, suggesting that while medical resources in the local area have improved significantly, it may create competitive pressure or a resource extraction effect on neighboring areas. This “siphoning effect” may weaken the economic resilience of surrounding regions as the concentration of medical resources often accompanies a unidirectional flow of talents and funds, which is not conducive to balanced regional economic development.
(6) Urban Environmental: Improvement not only directly enhances the economic resilience of the area but also positively affects the economic stability of neighboring areas by improving the quality of life and environmental attractiveness. This positive spillover effect indicates that high-quality regional environments are a win–win factor for regional development, capable of driving broad economic growth by enhancing the overall attractiveness and competitiveness of the area.

#### 4.2.3. Heterogeneity Analysis Based on Regional Differences

After conducting a comprehensive analysis of regional economic resilience across the country using the SDM, this study further investigates the differential impacts of various factors on economic resilience within the eastern, central, and western regions. As shown in Table 8, the three major regions display distinct heterogeneity in the factors affecting economic resilience, reflecting the unique characteristics and specific roles of regional factors across different parts of China. Taking the adjacency matrix as an example:

(1) Human Capital Level:

In the eastern region, human capital has a significant positive direct impact on economic resilience, likely due to the abundance of educational and training resources that foster innovation and economic activity. However, its indirect effect is negative, suggesting a “siphon effect” where areas with concentrated human capital may draw resources away from surrounding regions, negatively impacting them.

In the central region, neither the direct nor indirect effects of human capital are significant, which reflects the limitations in human resource development and utilization, indicating an ineffective transformation of human capital into economic resilience.

In the western region, the direct effect of human capital is significantly negative, and the indirect effect is not significant, suggesting issues with inadequate allocation and utilization efficiency of human resources, necessitating further policy support and resource optimization.

(2) Financial Development Level:

In the eastern region, both direct and indirect effects are positive, indicating that the region’s mature financial markets significantly contribute to regional economic stability and growth.

In the central region, financial development has a positive impact on economic resilience, suggesting that enhancements in financial services are gradually becoming a sustainable economic driver. However, the absence of significant indirect effects shows that financial development within the region has not yet resulted in spillover effects to stimulate surrounding areas.

In the western region, the direct effect of financial development is significantly negative, and the indirect effect is not significant, possibly due to the uneven level of financial development within the region and the concentration of financial centers in a few economically advanced cities, which does not benefit the region uniformly and exacerbates disparities.

(3) Market Consumption Level:

In the eastern and central regions, market consumption has significant positive direct and indirect effects on economic resilience, reflecting the well-developed market economies that have created large and diverse markets, thus positively influencing overall regional economic resilience.

In the western region, neither the direct nor indirect effects are significant, likely due to the region’s remoteness, relatively underdeveloped infrastructure, and local policies that focus more on stability and poverty alleviation than on market development, which restricts the positive impact of market consumption on economic resilience.

(4) Investment Openness Level:

In the eastern region, neither the direct nor indirect effects are significant due to the mature and open economic structure, where domestic and foreign investments are widespread, and the market is relatively saturated.

In the central region, the direct effect is significantly negative, and the indirect effect is not significant, reflecting that external investments have not effectively translated into actual productivity, and may have instead caused economic bubbles, negatively impacting regional economic resilience.

In the western region, the direct effect is not significant, and the indirect effect is significantly negative, suggesting that external investments may exacerbate the transfer of resources to more developed areas, with the benefits of investment being less than the losses caused by the outflow of talent resources, thereby reducing economic resilience.

(5) Urban Hospital Level:

In all three regions, the direct effects are significantly positive, indicating that medical levels have a uniformly positive impact, with developed medical services being crucial for enhancing economic resilience.

Indirect effects vary, with a negative impact in the eastern region, a non-significant impact in the central region, and a positive impact in the western region. This can be interpreted as differing levels of medical development across regions; in the east, high overall medical levels have created a siphon effect, drawing resources inward, a phenomenon not yet seen in the central and western regions.

(6) Urban Environmental Quality:

In the eastern region, the direct effect is significantly positive, but the indirect effect is significantly negative, suggesting that while improving urban environmental quality boosts the economic resilience of the area, it also acts as a siphon effect, drawing resources from other regions.

In the central region, the direct effect is significantly negative, and the indirect effect is significantly positive, indicating that while the regional environment contributes negatively to economic resilience internally, it positively impacts surrounding areas. This could be due to the economic restructuring from heavy industry to high-tech and service industries since 2009, which, despite improving environmental quality, has introduced instability affecting regional economic resilience.

In the western region, the direct effect is significantly positive, and the indirect effect is not significant, indicating a positive role of environmental quality in enhancing internal economic resilience without significant spillover effects to neighboring regions.

Overall, the eastern, central, and western regions of China show significant heterogeneity in factors affecting economic resilience. The eastern region’s factors align closely with national trends, with high levels of economic development and openness, placing it in a favorable position regarding economic resilience. The central region is experiencing rapid industrialization and urbanization, facing challenges from social and environmental aspects. The western region faces challenges in developing economic resilience due to geographical and infrastructural limitations but has potential for long-term improvement through policy support and resource optimization. These regional disparities highlight the need for policymakers to adopt targeted strategies to promote stability and development across different areas.

#### 4.2.4. Spatial and Temporal Evolution of Impact Factors

**Figure 4:**
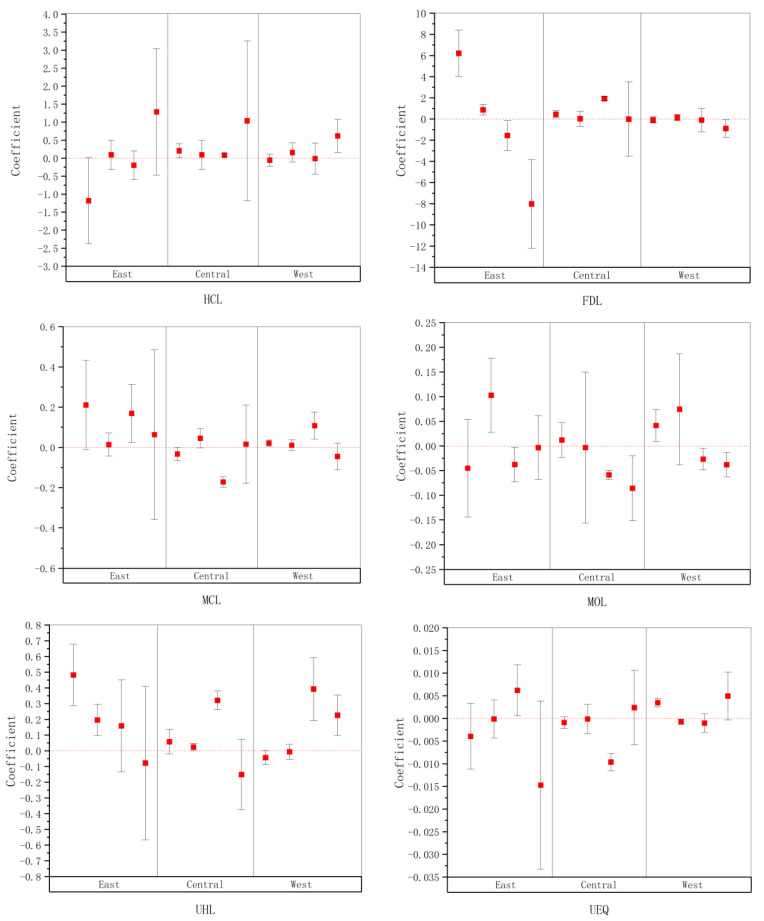
Temporal and spatial evolution of influencing factors. *Note*: The red dashed lines represent the reference line. The red boxes show the points of the regression coefficients, while the black lines indicate the confidence intervals.

To delve deeper into the evolution of impact factors across both spatial and temporal dimensions, this study selected four time intervals: 2000–2008, 2009–2015, 2016–2018, and 2019–2022. Using the SDM, we conducted empirical analyses on the factors affecting regional economic resilience in China, which yielded coefficients representing the contributions of these factors across different time periods. Chinese provinces were categorized into eastern, central, and western regions, and regression analyses were performed using adjacency matrices as an example, resulting in interval graphs (Figure 4) that depict the time-based changes in regression coefficients for each impact factor within the three major regions, highlighting their spatial and temporal evolution trends.

(1) Spatial and Temporal Divergence in the Impact of Human Capital on Economic Resilience

Throughout the sample period, the coefficients for human capital levels in the eastern, central, and western regions all show an upward trend, indicating that over time, the role of human capital in regional economic resilience has gradually strengthened. This change is primarily due to the Chinese government’s sustained investment in, and comprehensive reforms of, education and human resources policies. Notably, through the implementation of strategies like the Rise of Central China and the Development of the Western Regions, educational resources in the central and western regions have been significantly enhanced, and the vocational and higher education systems have also been expanded accordingly. Additionally, with the profound restructuring of the national economy, particularly the transition from traditional manufacturing to services and high-tech industries, the demand for highly skilled labor has markedly increased, enhancing the stature and importance of human capital within the economic structure.

Spatially, the eastern region exhibits the most significant change in human capital level coefficients, with a drastic fluctuation from negative to positive, indicating significant achievements in the effective utilization and rapid development of human capital in this region. The main reason behind this is the abundance of educational resources and a large talent pool in the eastern region. Alongside rapid economic development and quick changes in industrial structure, the existing vast human resources have been fully leveraged and utilized, shifting the talent market from an oversupply to a demand exceeding supply, thereby increasingly highlighting the positive impact of human capital on economic resilience. By contrast, although there has been progress in the central and western regions, their growth magnitude and speed are relatively slower. Particularly in the western region, due to a lower developmental starting point, changes in human capital coefficients are relatively minor, indicating that despite some policy support, issues of unbalanced development and a relative scarcity of educational and vocational training resources still persist. The central and western regions need to further enhance investments in human capital and improve their education and training systems to boost their economic resilience and autonomous capacity to handle external pressures.

(2) Spatial and Temporal Divergence in the Impact of Financial Development on Economic Resilience

During the sample period, the coefficients for financial development levels in the eastern, central, and western regions all exhibit varying degrees of decline, with the trend most pronounced in the eastern region and fluctuating downwards in the central and western regions. This phenomenon suggests that while financial development generally has a positive impact on economic resilience, its efficacy has diminished over time and even shown negative tendencies. This might be explained by the robust development of financial markets between 2000 and 2008, especially in the eastern region, which initially drove substantial economic growth. However, following the 2008 financial crisis, the government increased regulatory measures to prevent resource misallocation and financial bubbles, with these policies particularly evident in the eastern region.

Spatially, the impact of financial development levels on economic resilience displays significant regional differences between the eastern and central–western regions. In the eastern region, the coefficient of financial development impact initially decreased significantly from a positive value to a negative value, indicating that the rapid development of financial markets initially drove economic growth and complexity, but as the financial sector overheated and potential resource misallocation occurred, its development began to negatively affect economic resilience, necessitating regulatory adjustments to balance and maintain healthy development of financial markets and economic stability. In the central and western regions, the impact of financial development on economic resilience fluctuates around zero—weaker compared to the eastern region—due to relatively backward financial infrastructure and inadequate financial service coverage and depth, particularly evident in rural and remote areas distant from urban centers. Moreover, the economies in these regions rely heavily on traditional industries and low-value-added agriculture, limiting the demand for and stimulus from financial products and services innovation. Therefore, enhancing the prevalence and quality of financial services and promoting financial innovation adapted to local economic characteristics are key paths for improving the impact of financial development on economic resilience in the central and western regions.

(3) Spatial and Temporal Divergence in the Impact of Market Consumption on Economic Resilience

During the sample period, the impact of market consumption on economic resilience exhibits significant regional differences among the eastern, central, and western regions. The trends for the eastern and western regions are broadly similar, whereas the situation in the central region is distinctly different, showing the complementarity between the eastern and central regions. Against the backdrop of the domestic goods market as an organic whole, the market exhibits dynamic fluid characteristics. When the economic environment is stable, the eastern region, with its vast market consumption, acts as a major contributor to consumption, significantly and positively impacting economic resilience. However, as the eastern region is more closely linked to the global economy, it is more susceptible to economic crises. When the economy faces shocks, market activities gradually tilt toward the central region. During this shift, the central market begins to play a countercyclical role, displaying a positive impact on economic resilience. As the economy gradually stabilizes, market activities flow back to the eastern region, relatively weakening the market advantage of the central region, while the positive impact of the eastern region re-emerges.

Spatially, the vast market consumption in the eastern region continuously provides a wealth of economic activities and business opportunities, thereby maintaining a consistently positive overall impact on economic resilience. In contrast, the market consumption in the central region shows its positive role when the eastern region encounters economic shocks, indicating that the national development strategy for the central region is beginning to bear fruit, and the central region is able, to some extent, to accommodate the transferred consumption and production demands from the eastern region. The impact of market consumption on economic resilience in the western region exhibits volatility; due to relatively backward infrastructure and economic development levels, the economic influence of market consumption is unstable and easily affected by external economic environments.

(4) Spatial and Temporal Divergence in the Impact of Investment Openness on Economic Resilience

During the sample period, the impact of investment openness on economic resilience shows clear temporal changes. In the initial stage, the absorption and adaptation capacity for external investments vary among regions; hence, the impact of investment openness on economic resilience appears both positive and negative. However, as time progresses, particularly in the latter stages of the sample, this impact generally shifts toward the negative. This trend indicates that although external investments might initially promote regional economic growth through capital inflows and technology transfers, excessive reliance on external capital may increase the sensitivity of the regional economy, leading to global economic uncertainties being transmitted to the region through foreign investments, amplifying the vulnerability of the regional economy.

Spatially, the level of investment openness in the eastern region initially shows an upward trend then transitions to a decline, eventually predominantly displaying negative impacts. This phenomenon may stem from the high degree of openness in the regional economy; the massive influx of foreign capital initially drives rapid economic growth, but as reliance on foreign capital increases, external economic fluctuations begin to negatively impact the region, necessitating adjustments and regulations to balance and maintain the health of financial markets and economic stability. Although the initial impacts in the central region exhibit some positive effects, these effects gradually diminish and turn negative, reflecting the intrinsic limitations of the region in attracting foreign investments and converting them into economic momentum. The situation in the western region is more complex; although the positive impacts of investment openness are initially quite apparent, due to insufficient infrastructure and industrial foundations, these positive effects are not sustainable and quickly turn negative, revealing the challenges of the western region in continuously utilizing foreign investments to stimulate economic growth.

(5) Spatial and Temporal Divergence in the Impact of Urban Hospital Level on Economic Resilience

During the sample period, the impact of urban hospital levels on economic resilience shows significant spatial and temporal divergence. In the initial period, the high medical levels in the eastern and central regions significantly promote economic resilience, reflecting the positive role of good medical facilities and service quality in improving residents’ health and quality of life, enhancing the efficiency of economic activities, and boosting the overall resilience of regional economies. However, over time, particularly in the latter stages of the sample period, the impact of these urbans’ hospital levels on economic resilience gradually turns negative, with a possible explanation being the uneven distribution of medical resources and the diminishing marginal benefits.

Spatially, the urban hospital levels in the eastern and central regions initially have a significant positive impact on economic resilience, but both exhibit negative impacts in the later stages. This change may be related to increasing population density in these regions and the consequent demand for medical resources, particularly during the final stage, affected by the COVID-19 shock, which significantly increased the demand for medical resources in all regions, severely straining the medical system. This impact is especially pronounced in the densely populated eastern and central regions. In contrast, although initially weaker in the western region, the positive impact becomes significant in the latter stages of the observation period, likely due to the government’s recent efforts to strengthen medical infrastructure and service quality in the region, effectively improving the health status and economic adaptability of local residents.

(6) Spatial and Temporal Divergence in the Impact of Urban Environmental Quality on Economic Resilience

During the sample period, the impact of urban environmental quality on economic resilience generally shows an upward trend, only declining in the eastern region during the final stage, indicating that as society develops, public concern for the ecological environment increases. Coupled with the continuous strengthening of government environmental policies and sustained increases in green investments in recent years, the positive association between urban environmental quality and economic resilience has gradually strengthened.

Spatially, the initial period shows a consistently positive association between environmental quality and economic resilience in the eastern region, with only the final stage displaying a negative impact, mainly due to the economic resilience decline caused by the pandemic shock in the eastern region. Environmental governance, as a long-term project, is not directly affected by the pandemic shock. Although the central region shows a negative impact of environmental quality on economic resilience for most of the period, the improvement in the final stage reflects the initial success of environmental governance strategies. The situation in the western region is more positive, especially in the latter part of the sample period, where the improvement in environmental quality significantly positively impacts economic resilience. This may be attributed to the region’s continuous investment in environmental protection and natural resource management.

## 5. Discussion

Based on data from 31 Chinese provinces from 2000 to 2022, this study investigates the spatiotemporal dynamics and influencing factors of regional economic resilience, utilizing the SDM for empirical analysis. The findings reveal that regional economic resilience in China exhibits significant spatial clustering, suggesting that resilience is not only closely related to local economic activities but also influenced by neighboring regions. From a spatiotemporal perspective, economic resilience shows different dynamic changes across the eastern, central, and western regions. The eastern region demonstrates notably stronger economic resilience than other areas, with resilience closely linked to regional development strategies and external factors.

First, this study finds significant spatial clustering of economic resilience, indicating that resilience is influenced not only by local economic activities but also by spatial spillover effects from neighboring regions. This result aligns with the findings of Giannakis et al., who studied regional economies within the European Union [63]. Moreover, this study further reveals the spatiotemporal heterogeneity of influencing factors, expanding our understanding of the spatiotemporal distribution of economic resilience.

Second, from a regional perspective, while regional economic resilience in China has increased over the years, its dynamic changes in the eastern, central, and western regions differ significantly, which is consistent with previous research [64]. Overall, economic resilience exhibits a “high in the east, low in the west” spatially uneven distribution, with the eastern coastal regions showing significantly higher resilience than the inland areas of the central and western regions. Over time, the east–west disparity has become more pronounced, indirectly reflecting the uneven development between eastern and western China. However, it should be noted that some scholars argue that under different economic shock conditions, the performance of regional economic resilience across provinces is not entirely uniform [65].

Third, kernel density estimation results show that economic resilience has improved to varying degrees nationwide and in the three major regions over time, yet regional disparities have not been effectively reduced. This finding aligns with the conclusions of Li and Liu on the economic resilience of resource-based cities in China [66]. Further breakdown by region shows that the eastern region has been closing its gaps, with internal disparities gradually decreasing; the central region has shown an overall upward trend, with more balanced development; while the western region has seen minimal change, though the highest resilience levels continue to improve.

Empirical results indicate that the effects of various factors on economic resilience differ, and these factors exhibit significant spatial and temporal heterogeneity. Specifically, human capital and healthcare levels have a direct positive effect on resilience within the region, which is consistent with existing research [57,67,68]. This study further finds that these factors have a negative indirect effect on neighboring regions, with a significant spatial siphon effect. Financial development, market scale, and environmental quality exhibit clear spatial spillover effects, positively promoting economic resilience both within the region and in surrounding regions, expanding upon previous studies [55,56]. From a spatial perspective, the effects of these factors in the eastern region align with national trends, while the central and western regions show distinct differences. For instance, human capital has no significant direct or indirect effects in the central region, and even shows a negative direct effect in the western region. Temporally, the spatiotemporal evolution of these influencing factors varies across regions. For example, in the eastern region, the influence of healthcare levels has been declining, while in the western region, it has shown a continuous upward trend.

## 6. Policy Recommendations

First, strengthening investment in human capital, especially in education and vocational skills in the central and western regions, is a fundamental policy direction for enhancing regional economic resilience. Improving the quality of education and expanding vocational training opportunities are key to enhancing the workforce’s skills, thereby boosting economic resilience. The results of this study show that the positive impact of human capital on economic resilience is more pronounced in the eastern region, while improving human capital levels is particularly critical in the central and western regions. Therefore, the government should focus on optimizing the allocation of educational resources, particularly in remote areas, to improve the overall quality of the workforce and thereby strengthen economic resilience.

Second, promoting the modernization and popularization of financial services to improve capital liquidity is essential. The findings of this study indicate that financial development has significant spatial spillover effects on regional economic resilience, especially in the eastern region. Therefore, it is recommended to further modernize financial services, particularly in the central and western regions, by strengthening financial infrastructure and increasing the accessibility of financial services to improve service efficiency. This policy will not only help capital flow more effectively but will also promote the rational allocation of resources, supporting local economic development. In particular, in the central and western regions, financial support can help local businesses access more financing opportunities, thereby enhancing their resilience to economic shocks.

Third, expanding market scale and optimizing the business and investment environment is vital for promoting market development. The government should encourage the expansion of market size and business activity through policy incentives and infrastructure investment. Simultaneously, the investment environment should be continuously optimized by simplifying investment procedures and enhancing regional attractiveness for foreign and private investments. Based on the findings of this study, the financial and service sectors in the eastern region already show strong resilience, so market access for high-end manufacturing and modern service industries can be further relaxed in this area. In contrast, in the central and western regions, the government should attract more industrial transfer and technological upgrading projects by offering tax incentives, land policy support, and other measures, thereby driving regional economic transformation and improving resilience.

Fourth, enhancing infrastructure in the central and western regions, particularly healthcare services and green industries, is crucial. This study also highlights the significant impact of healthcare levels and environmental quality on economic resilience. In the central and western regions, the government should increase investments in regional infrastructure, particularly in healthcare facilities, to improve accessibility and quality of healthcare resources. This will not only enhance public health but also boost the region’s economic resilience. Additionally, the government should vigorously promote sustainable urban planning and green building practices, improve air and water quality, and enhance the overall living environment. These measures will not only improve residents’ quality of life but also increase the region’s competitiveness and attractiveness.

Fifth, considering the regional disparities, policy design should adopt differentiated approaches and strengthen coordination and cooperation between regions. Given the “strong east, weak west” economic distribution in China, policies should be customized and regionally tailored to maximize their effectiveness. In particular, in the central and western regions, which have lower economic resilience, policies can focus on infrastructure development and industrial transformation to boost economic growth and resilience. Additionally, strengthening inter-regional coordination and cooperation, utilizing the comparative advantages of different regions, and forming a complementary economic development model are key to enhancing national economic resilience. Collaborative efforts between regions can promote optimal resource allocation and support balanced, nationwide economic development.

Despite the comprehensive analysis of the spatiotemporal evolution and influencing factors of regional economic resilience in China, this study has certain limitations. On one hand, the study is based on provincial-level data, which may not fully capture the finer-grained heterogeneity within regions. Future research could expand to the city-level to obtain more detailed dynamic information. On the other hand, this study does not include international comparative analysis. Future research could incorporate case studies from other countries to explore the commonalities and differences in economic resilience. Moreover, this study primarily analyzes economic resilience from a cross-sectional perspective and does not delve into the dynamic impact of policy interventions or external shocks (such as the pandemic) on resilience. Future studies could use dynamic models to explore the underlying mechanisms of resilience changes. These improvements would further enrich the understanding of regional economic resilience and provide valuable references for future research.

## Figures and Tables

**Table 1:** China’s economic resilience indicator system.

Level 1 Indicator	Level 2 Indicator	Level 3 Indicator	Unit	Expected Direction
Regional Economic Resilience	Economic Resistance Resilience	Per Capita Regional GDP	(Yuan/person)	Positive
		Per Capita Disposable Income of All Residents	(Yuan)	Positive
		Savings Balance of All Residents	(Yuan)	Positive
		Urban Registered Unemployment Rate	(%)	Negative
		Foreign Trade Dependence	(%)	Negative
	Economic Recovery Resilience	Tertiary Industry’s Share of GDP	(%)	Positive
		Regional GDP Index	(%)	Positive
		Advancement of Industrial Structure	(%)	Positive
		Total Retail Sales of Consumer Goods	(Billion Yuan)	Positive
		Local Fiscal Revenue and Expenditure Ratio	(%)	Positive
	Economic Reorganization Resilience	Number of Domestic Patent Applications Granted	(Items)	Positive
		R&D Spending as a Percentage of GDP	(%)	Positive
		Local Government Spending on Education	(Billion Yuan)	Positive
		Local Government Spending on Science and Technology	(Billion Yuan)	Positive
		Number of Students in Regular Higher Education Institutions	(Ten thousand people)	Positive

**Table 2:** Variable definitions.

Category	Indicator	Definition	Calculation Method
Regional Development	Human Capital Level (HCL)	The reserve of labor force in the region	Number of people aged 15–64 (from population sampling survey) (persons)/Total population (from population sampling survey) (persons)
	Financial Development Level (FDL)	The development of financial institutions and the prevalence of financial services in the region	Added value of the financial sector (Billion Yuan)/GDP
Market Development	Market Consumption Level (MCL)	The capacity and consumption potential of the regional market	The logarithm of total retail sales of consumer goods (Billion Yuan)
	Investment Openness Level (MOL)	The degree of openness to foreign investment in the region	Amount of foreign investment by foreign-invested enterprises in the current year (Billion Yuan)/GDP
Regional Development	Urban Hospital Level (UHL)	The development level of the public health system and the quality of medical services in the region	The logarithm of the number of hospital beds (units)
	Urban Environmental Quality (UEQ)	The quality of greening, air quality, and water resource management in the region	Green coverage rate in built-up areas (%)

**Table 3:** Variable descriptions.

Category	Indicator Name	N	Mean	SD	Min	Max	p50
Regional Development	Human Capital Level	713	0.722	0.037	0.647	0.813	0.719
	Financial Development Level	713	0.057	0.031	0.016	0.18	0.052
Market Development	Market Consumption Level	713	8.1	1.337	4.5	10.537	8.246
	Investment Openness Level	713	0.058	0.109	−0.201	0.683	0.028
Regional Development	Urban Hospital Level	713	2.679	0.81	0.56	4.189	2.774
	Urban Environmental Quality	713	35.882	5.984	18.1	49.8	37.3
Regional Economic Resilience (RES)		713	0.199	0.166	0.024	0.688	0.155

**Table 4:** Global Moran’s indices (using adjacency matrix).

Year	I	E(I)	sd(I)	z	*p*-Value
2000	0.340	−0.033	0.115	3.244	0.001
2001	0.329	−0.033	0.114	3.167	0.002
2002	0.311	−0.033	0.115	3.006	0.003
2003	0.304	−0.033	0.114	2.944	0.003
2004	0.317	−0.033	0.115	3.036	0.002
2005	0.320	−0.033	0.116	3.052	0.002
2006	0.331	−0.033	0.116	3.131	0.002
2007	0.317	−0.033	0.116	3.016	0.003
2008	0.306	−0.033	0.117	2.913	0.004
2009	0.345	−0.033	0.117	3.243	0.001
2010	0.354	−0.033	0.117	3.320	0.001
2011	0.363	−0.033	0.117	3.395	0.001
2012	0.368	−0.033	0.117	3.435	0.001
2013	0.359	−0.033	0.117	3.354	0.001
2014	0.382	−0.033	0.117	3.543	0.000
2015	0.366	−0.033	0.117	3.422	0.001
2016	0.365	−0.033	0.117	3.414	0.001
2017	0.343	−0.033	0.116	3.236	0.001
2018	0.345	−0.033	0.116	3.252	0.001
2019	0.349	−0.033	0.116	3.287	0.001
2020	0.383	−0.033	0.117	3.568	0.000
2021	0.420	−0.033	0.117	3.862	0.000
2022	0.392	−0.033	0.117	3.625	0.000

**Table 5:** Test results for selection of spatial econometric models.

Spatial Panel Model Tests			Adjacency Matrix		Geographic Distance Matrix	
			Statistic	*p*-Value	Statistic	*p*-Value
LM Test	Spatial lag	Moran’s I	3.575	0.000	1.80 × 10^5^	0.000
		LM-test-lag	229.082	0.000	120.29	0.000
		Robust LM-test-lag	237.472	0.000	70.633	0.000
	Spatial error	LM-test-error	22.405	0.000	60.818	0.000
		Robust LM-test-error	30.795	0.000	11.161	0.001
Individual/Time/Both Fixed Effects	LR-both/ind		122.87	0.000	69.09	0.000
	LR-both/time		594.74	0.000	635.91	0.000
LR Test	LR-SDM/SAR		100.57	0.000	71.92	0.000
	LR-SDM/SEM		108.06	0.000	80.79	0.000
Wald Test	Wald-SDM/SAR		108.28	0.000	74.63	0.000
	Wald-SDM/SEM		115.27	0.000	84.35	0.000
Hausman Test	Hausman		101.41	0.000	101.41	0.000

**Table 6:** Model regression results.

	OLS	Adjacency Matrix	Geographic Distance Matrix
	Resilience	Resilience	Resilience
HCL	0.0545	0.424 ***	0.272 **
	(0.35)	(3.07)	(1.97)
FDL	1.561 ***	0.925 ***	1.101 ***
	(6.49)	(4.47)	(5.17)
MCL	0.00951	0.0179	0.0172
	(0.65)	(1.23)	(1.16)
MOL	−0.0220	−0.00657	−0.0211
	(−1.02)	(−0.35)	(−1.15)
UHL	0.0973 ***	0.138 ***	0.144 ***
	(3.93)	(5.94)	(6.14)
UEQ	0.00810 ***	0.00493 ***	0.00474 ***
	(8.83)	(6.01)	(5.73)
W×HCL		−1.715 ***	−0.276
		(−7.87)	(−0.73)
W×FDL		1.809 ***	3.535 ***
		(4.36)	(7.49)
W×MCL		0.0280	−0.00970
		(1.30)	(−0.32)
W×MOL		−0.0525	−0.0220
		(−1.26)	(−0.45)
W×UHL		−0.119 ***	−0.0505
		(−2.68)	(−0.96)
W×UEQ		0.00441 ***	0.000217
		(3.13)	(0.11)
Spatial		0.376 ***	0.506 ***
rho		(9.27)	(9.97)
Variance		0.00204 ***	0.00208 ***
sigma2_e		(18.65)	(18.30)
r2	0.7306	0.347	0.326
N	713	713	713

*Note:* *, **, *** indicate significance at the 10%, 5%, and 1% levels, respectively, with t-values in parentheses.

**Table 7:** Spatial effect decomposition.

	Adjacency Matrix			Geographic Distance Matrix		
	Direct Effect	Indirect Effect	Total Effect	Direct Effect	Indirect Effect	Total Effect
HCL	0.269 *	−2.345 ***	−2.075 ***	0.262 *	−0.261	0.00183
	(1.89)	(−6.60)	(−5.11)	(1.70)	(−0.34)	(0.00)
FDL	1.133 ***	3.201 ***	4.334 ***	1.526 ***	7.799 ***	9.325 ***
	(5.40)	(5.50)	(6.29)	(6.80)	(6.95)	(7.55)
MCL	0.0229 *	0.0540 *	0.0769 **	0.0186	0.000534	0.0192
	(1.67)	(1.79)	(2.35)	(1.32)	(0.01)	(0.34)
MOL	−0.0122	−0.0803	−0.0924	−0.0245	−0.0578	−0.0823
	(−0.63)	(−1.24)	(−1.24)	(−1.27)	(−0.60)	(−0.78)
UHL	0.130 ***	−0.109 *	0.0215	0.145 ***	0.0313	0.176 *
	(5.73)	(−1.71)	(0.31)	(6.08)	(0.31)	(1.65)
UEQ	0.00560 ***	0.00955 ***	0.0151 ***	0.00505 ***	0.00518	0.0102 **
	(6.81)	(4.62)	(6.33)	(5.91)	(1.38)	(2.48)
r2	0.347			0.326		
N	713			713		

*Note*: *, **, *** indicate significance at the 10%, 5%, and 1% levels, respectively, with t-values in parentheses.

**Table 8:** Regional differences.

	Adjacency Matrix			Geographic Distance Matrix		
	Eastern Region	Central Region	Western Region	Eastern Region	Central Region	Western Region
HCL	0.787 ***	0.00863	−0.348 ***	0.711 ***	0.0906	−0.283 ***
	(2.95)	(0.09)	(−3.93)	(2.76)	(0.88)	(−3.51)
FDL	1.462 ***	0.770 ***	−0.273 **	2.536 ***	0.758 ***	−0.128
	(3.21)	(4.76)	(−2.34)	(5.16)	(4.94)	(−1.22)
MCL	0.118 ***	0.0203 **	0.00586	0.0751 **	0.0247 ***	0.00781
	(4.07)	(2.08)	(0.80)	(2.56)	(2.75)	(1.14)
MOL	−0.0153	−0.0310 **	−0.0130	−0.0264	−0.0398 ***	−0.0204 *
	(−0.56)	(−2.16)	(−1.02)	(−0.97)	(−2.84)	(−1.77)
UHL	0.125 ***	0.0906 ***	0.0976 ***	0.211 ***	0.0864 ***	0.0698 ***
	(2.65)	(6.05)	(8.24)	(4.43)	(5.95)	(6.29)
UEQ	0.00320	−0.00106 *	0.00163 ***	−0.00410	−0.000653	0.00130 ***
	(1.35)	(−1.83)	(3.86)	(−1.64)	(−1.16)	(3.54)
W×HCL	−1.240 ***	0.289 *	−0.127	−0.790	0.650 ***	−0.651 **
	(−3.67)	(1.69)	(−0.62)	(−1.33)	(2.99)	(−2.30)
W×FDL	1.468 *	−0.166	−0.407	1.496 *	−0.354	−0.560 *
	(1.89)	(−0.59)	(−1.49)	(1.93)	(−1.14)	(−1.68)
W×MCL	0.114 ***	0.0186	−0.0110	−0.0427	0.0214 *	0.00735
	(2.71)	(1.56)	(−0.61)	(−0.71)	(1.68)	(0.25)
W×MOL	−0.0685	−0.0200	−0.103 ***	−0.0349	−0.0369 **	−0.127 ***
	(−1.40)	(−1.28)	(−3.33)	(−0.58)	(−2.10)	(−3.57)
W×UHL	−0.119	0.0184	0.0704 **	−0.120	0.0124	0.0602 *
	(−1.57)	(0.73)	(2.37)	(−1.05)	(0.43)	(1.74)
W×UEQ	−0.0120 ***	0.00300 ***	0.00155	−0.0198 ***	0.00375 ***	0.00552 ***
	(−2.74)	(3.81)	(1.50)	(−5.14)	(4.41)	(4.29)
Spatial	−0.154 **	0.0184	−0.183 *	−0.362 ***	−0.0770	0.0137
rho	(−2.41)	(0.23)	(−1.88)	(−4.27)	(−0.83)	(0.13)
Variance	0.00261 ***	0.000119 ***	0.000210 ***	0.00245 ***	0.000111 ***	0.000175 ***
sigma2_e	(11.19)	(9.59)	(11.65)	(10.98)	(9.56)	(11.75)
LR_Direct						
HCL	0.889 ***	0.0144	−0.341 ***	0.840 ***	0.0736	−0.281 ***
	(3.17)	(0.14)	(−3.73)	(2.79)	(0.72)	(−3.35)
FDL	1.355 ***	0.764 ***	−0.261 **	2.460 ***	0.767 ***	−0.134
	(3.09)	(4.87)	(−2.35)	(5.26)	(5.21)	(−1.32)
MCL	0.114 ***	0.0214 **	0.00694	0.0855 ***	0.0249 ***	0.00844
	(4.06)	(2.27)	(0.99)	(2.97)	(2.79)	(1.27)
MOL	−0.0114	−0.0314 **	−0.00870	−0.0241	−0.0390 ***	−0.0208 *
	(−0.42)	(−2.24)	(−0.69)	(−0.90)	(−2.88)	(−1.85)
UHL	0.132 ***	0.0908 ***	0.0952 ***	0.232 ***	0.0862 ***	0.0696 ***
	(2.89)	(6.16)	(8.02)	(4.97)	(5.92)	(6.46)
UEQ	0.00407 *	−0.00101 *	0.00159 ***	−0.00201	−0.000751	0.00133 ***
	(1.83)	(−1.76)	(3.90)	(−0.85)	(−1.35)	(3.53)
LR_Indirect						
HCL	−1.287 ***	0.294	−0.0561	−0.894 *	0.622 ***	−0.663 **
	(−3.95)	(1.62)	(−0.30)	(−1.68)	(2.85)	(−2.32)
FDL	1.129 *	−0.162	−0.320	0.454	−0.407	−0.570 *
	(1.76)	(−0.62)	(−1.38)	(0.76)	(−1.52)	(−1.69)
MCL	0.0914 **	0.0192 *	−0.00971	−0.0562	0.0187	0.00998
	(2.27)	(1.65)	(−0.64)	(−1.15)	(1.49)	(0.35)
MOL	−0.0605	−0.0200	−0.0881 ***	−0.0201	−0.0317 *	−0.129 ***
	(−1.31)	(−1.26)	(−3.13)	(−0.40)	(−1.92)	(−3.42)
UHL	−0.135 **	0.0171	0.0439 *	−0.174 *	0.00209	0.0579 *
	(−2.03)	(0.71)	(1.75)	(−1.94)	(0.08)	(1.83)
UEQ	−0.0113 ***	0.00308 ***	0.00117	−0.0153 ***	0.00371 ***	0.00572 ***
	(−2.65)	(3.61)	(1.22)	(−4.54)	(4.23)	(4.06)
LR_Total						
HCL	−0.398	0.308	−0.397 *	−0.0549	0.695 **	−0.943 ***
	(−1.33)	(1.27)	(−1.96)	(−0.13)	(2.49)	(−3.12)
FDL	2.484 ***	0.602 *	−0.581 **	2.914 ***	0.359	−0.704 **
	(3.18)	(1.86)	(−2.22)	(3.73)	(1.15)	(−2.12)
MCL	0.206 ***	0.0406 ***	−0.00278	0.0293	0.0436 ***	0.0184
	(4.84)	(4.31)	(−0.16)	(0.55)	(4.49)	(0.59)
MOL	−0.0719	−0.0514 **	−0.0968 ***	−0.0443	−0.0706 ***	−0.149 ***
	(−1.39)	(−2.08)	(−3.46)	(−0.84)	(−2.94)	(−3.67)
UHL	−0.00304	0.108 ***	0.139 ***	0.0577	0.0883 ***	0.128 ***
	(−0.04)	(4.18)	(5.54)	(0.61)	(3.35)	(3.66)
UEQ	−0.00726	0.00207 **	0.00276 **	−0.0173 ***	0.00296 ***	0.00705 ***
	(−1.45)	(1.97)	(2.47)	(−4.13)	(2.86)	(4.52)
r2	0.483	0.685	0.425	0.213	0.736	0.488
N	253	184	276	253	184	276

*Note*: *, **, *** indicate significance at the 10%, 5%, and 1% levels, respectively, with t-values in parentheses.

## Data Availability

The data presented in this study are available on request from the corresponding author.
